# Genome-wide identification and evolutionary analysis of SUT genes reveals key regulators of drought stress response in finger millet (*Eleusine coracana*)

**DOI:** 10.1016/j.jgeb.2025.100592

**Published:** 2025-10-14

**Authors:** Kasinathan Rakkammal, Pandiyan Muthuramalingam, Hyunsuk Shin, Manikandan Ramesh

**Affiliations:** a Department of Biotechnology, Science Campus, Alagappa University, Karaikudi 630003 Tamil Nadu, India; b Department of GreenBio Science, College of Agriculture and Life Sciences, Gyeongsang National University, Jinju 52728, Republic of Korea

**Keywords:** Sucrose transporter, *Eleusine coracana*, Drought stress, Gene expression, Phylogenetic analysis, Synteny analysis, Finger millet

## Abstract

Sucrose transporters (SUTs) mediate sucrose movement across plant membranes, playing a crucial role in carbon allocation and stress responses. Although finger millet (*Eleusine coracana*) is known for its inherent drought resistance, the specific involvement of *SUT* genes in this characteristic is still unclear. This study aimed to identify the *SUT* genes of millet and to assess their expression in drought conditions. Five *SUT* genes (*EcSUT1*-*EcSUT5*) were identified that encode proteins with 9–12 transmembrane domains. Phylogenetic analysis clustered these SUT members across all three main SUT groups, suggesting an evolutionary divergence within the family. Synteny analysis revealed conserved genomic regions, with *EcSUT2* showing 91–94% identity with orthologs in closely related grasses. Structural models further confirmed their typical transmembrane architecture. Interaction analysis identified EcSUT2 as a key interaction with SWEET transporters. Furthermore, the promoter regions of *EcSUT2* and *EcSUT5* were found to be enriched with hormone and stress-responsive elements. Under drought conditions, *EcSUT1-EcSUT4* displayed transient induction, while *EcSUT5* showed sustained upregulation, especially in the roots, notably after 48 h. The finger millet SUT family exhibits evolutionary conservation within grasses, with individual genes that play different roles in stress response. The persistent upregulation of *EcSUT5* under drought strongly suggests its involvement in maintaining sucrose transport during long-term adverse conditions. This candidate gene requires further functional validation to uncover the stress dynamics for sustainable crop improvement.

## Graphical abstract

**Figure:**
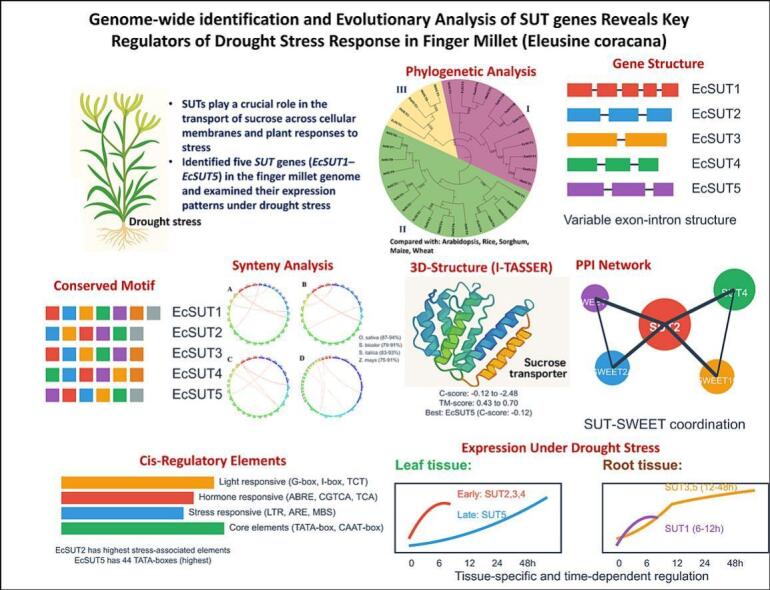

## 1 Introduction

Sucrose is a key molecule in plant metabolism, as a primary product of photosynthesis, an important signaling molecule, and an energy source that maintains a wide array of physiological processes. Sucrose transporters (*SUTs*), members of the major facilitator superfamily (MFS) of H^+^/sucrose symporters, mediate efficient sucrose translocation from source tissues (e.g., mature leaves) to sink tissues (e.g., roots, seeds, and developing organs). *SUTs* have a major function in phloem loading and unloading and cellular sugar homeostasis.,^1^, ^2^ *SUTs* are also responsive to abiotic stresses, hormones, and environmental signals, which highlights their key functions in plant development and stress physiology.^3^

Studies in other plant systems have shown that *SUT* expression tends to change in response to drought, reflecting their involvement in physiological adjustments. In rice, for instance, *OsSUT2*, a tonoplast-targeted transporter, is involved in mobilizing sucrose stored in vacuoles, perhaps to help osmotically regulate.^4^ *OsSUT1*, a plasma membrane-targeted protein, is needed for phloem loading and is expressed in tissue-specific expression that may be modulated in response to stress.^5^, ^6^ Similar responses are observed in tomato, where *SlSUT1* activity is up-regulated under drought, perhaps to sustain carbohydrate supply to the roots and retard leaf senescence.^7^ In tobacco, increased expression of *NtSUT1* correlates with enhanced export of sucrose from the leaf,^8^ while in barley, *HvSUT2* is thought to support root performance under limited water availability.^9^ Recent studies indicate that water deficit increases sucrose exports to roots in Arabidopsis through the co-ordination of *SUTs* between shoots and roots.^10^, ^11^

Finger millet (*Eleusine coracana* L. Gaertn) is an annual grass belonging to Poaceae family. It is cultivated mainly in the arid and semi-arid zones of Asia and Africa.^12^ It is the world's fourth most significant millet after sorghum, pearl millet, and foxtail millet^13^, ^14^ . Notably, finger millet has been recognized as a “super cereal,” finger millet is highly regarded for its remarkable nutritional profile. It contains a calcium content of approximately 0.38 %, a dietary fiber level of around 18 %, and a phenolic compound concentration ranging from 0.3 % to 3 % all of which are significantly higher than those found in staple cereals like rice, wheat, and maize. 15, 16 Its nutrient content and ability to grow in low-input, marginal environments make it an excellent crop to address future food and nutritional security concerns.

Genome-wide studies have enhanced knowledge about the *SUT* gene family in plants. However, sucrose transporters were identified and characterized in various plant species. Genome-wide identification and functional characterization of *SUT* genes in finger millet are yet to be explored. Therefore, the present study aims to identify and characterize the SUT gene family in the *E. coracana* genome and examine their expression patterns under drought stress. Our results might provide insight into the molecular importance of *EcSUTs* under drought stress. This study will be helpful for the identification and characterization of endogenous genes governing abiotic stress tolerance in finger millet.

## 2 Materials and methods

### 2.1 Identification and physicochemical characterization of *EcSUT* genes in finger millet

To identify sucrose transporter (*SUT*) genes in finger millet (*E. coracana*), protein sequences of the known *SUTs* of *Arabidopsis thaliana* and *O. sativa* were used as query sequences for BLASTP searches against the *E. coracana* genome, which is present in the Phytozome database (https://phytozome-next.jgi.doe.gov/info/Ecoracana_v1_1).^17^ Potential EcSUT candidates were selected based on the E value (E = 0) and 90 % sequence similarity. The coding sequence (CDS), peptide sequence, and genomic data of the identified genes were obtained for further analysis. Physical chemical properties such as amino acid length, molecular weight (MW), and theoretical isoelectric point (pI) have been calculated using ExPASy (https://expasy.org/protparam/).^18^ Predictions of subcellular localization were made through the Plant-mPLoc online tool (https://www.csbio.sjtu.edu.cn/bioinf/plant-multi/),^19^ whereas transmembrane domains were predicted by TMHMM Server v2.0 (https://www.cbs.dtu.dk/services/TMHMM-2.0/).^20^ Potential phosphorylation sites were predicted through the NetPhos 3.1 server (https://www.cbs.dtu.dk/services/NetPhos/),^21^ providing information on the structural and functional features of EcSUT proteins.

### 2.2 Phylogenetic and structural characterization of EcSUT genes

Multiple sequence alignment of EcSUT proteins with known SUTs from *A. thaliana* and *O. sativa* was carried out using Clustal Omega.^22^ A phylogenetic tree was constructed using MEGA X^23^ by the Neighbor-Joining (NJ) method with 1,000 bootstrap replicates, by the JTT model, and pairwise deletion. The tree was visualized with iTOL.^24^ Gene structures were then visualized using GSDS 2.0^25^, following alignment of coding sequences with their respective genomic DNA. Conserved motifs in the *EcSUT* proteins were identified using MEME Suite,^26^ with the motif number limited to 10 and widths set between 6 and 50 amino acids.

### 2.3 Tertiary structure prediction, protein-protein interaction, *cis*-regulatory element analysis, and synteny analysis

Three-dimensional EcSUT structures were predicted using I-TASSER, a template-based modeling approach to predict valid structural models.^27^ Structural conservation across isoforms was examined using PyMOL for visualization, aligning the predicted protein structures.^28^ In the PlantCARE database, a 1.5 kb upstream and 0.5 kb downstream promoter sequence was analyzed to identify the elements that act on *cis* associated with hormonal, environmental and developmental responses.^29^ The chromosome collinearity of the SUT genes of *E. coracana* and their orthologs of *O. sativa*, *Sorghum bicolor*, *Setaria italica*, and *Zea mays* are obtained from each genome annotation file. Syntenic relationships were illustrated using Circos (v0.69–9), a circular visualization tool designed for comparative genomics.^30^

### 2.4 Plant material, stress treatment, and gene expression analysis

This study used the drought-tolerant finger millet IC 87469 plant from ICAR-NBPGR in New Delhi. We gently transferred seedlings from 10 days old uniformly into hydroponic pots filled with a Hoagland half-strength solution. The plants were grown under controlled conditions of 25 °C ± 2°C and 70 % humidity, with a 16-hour light and a dark cycle of 200 µmol photons m^−2^s^−1^. Drought stress was induced by adding 20 % polyethylene glycol (PEG) to the nutrient solution of plants aged 14 days, while untreated plants served as control. Leaf (uppermost fully expanded leaves) and root tissues were collected at various time points: 0, 6, 12, 24, and 48 h after treatment, with three biological replicates for each time point. The 0-hour time point corresponded to untreated plants and was considered the control for all subsequent gene expression comparisons.

RNA was extracted from these samples by using the Trizol method. Subsequently, 500 ng of total RNA was reverse-transcribed to cDNA by QuantiTect Reverse Transcription Kit (Qiagen, Germany). The reaction was performed according to the kit instructions to ensure high-quality and reproducible cDNA synthesis. Afterward, quantitative real-time PCR was performed by using SYBR Green Rox, with the optimized cycling conditions for specificity and efficacy. The gene expression levels of *EcSUTs* were normalized with the housekeeping gene *EcEF1α*, and relative changes in gene expression were estimated by the 2^-ΔΔCt^ method.^31^ The *EcSUTs* and *EcEF1α*- primers used in the experiments were synthesized commercially, and their sequences are listed in Table S1.

### 2.5 Statistical analysis

All the experiments were completed in a perfectly randomized design with a minimum of three replicates. The collected data were examined using one-way analysis of variance (ANOVA), and the significance of changes within means was assessed at *P* ≤ 0.05 using Duncan's multiple range test with SPSS Statistics Software 17.0. The values in the figures are based on the mean values ± SD of three independent experiments.

## 3 Results

### 3.1 Physicochemical properties and phosphorylation patterns of EcSUTs

Five sucrose transport genes (*EcSUT1–EcSUT5*) were identified, all encoding proteins located on the cell membrane with 9 to 12 transmembrane helices. These genes are present in the 2A, 3A, and 5A chromosomes, and protein lengths range from 497 to 602 amino acids, with molecular weights ranging from 53.1 to 64 kDa. The isoelectric points (5.91–8.94), the instability index, and the GRAVY values are largely stable and hydrophobic (Table 1). The prediction of phosphorylation site showed that *EcSUT4* had the largest number of modifications, while *EcSUT1* had the smallest. In particular, *EcSUT3* and *EcSUT4* had the largest number of PKC sites, and all proteins were predicted to have cdc2 and CKII kinase sites. Unspecified kinase sites were particularly abundant in *EcSUT4* and *EcSUT5*, highlighting potential regulatory complexity among these transporters (Table 2).

**Table 1:** Identification and characterization of *EcSUT*s in finger millet.

Gene Name	Accession ID	Chromosomal location and strand (±)	Amino acid residues number	MW (kDa)	pI	Instability Index	Aliphatic Index	GRAVY	Subcellular location	Predicted TMHs	Amino acids in TMHs
*EcSUT1*	ELECO.r07.3AG0235420.1	3A:38174213..38179358 reverse	523	55.36	8.78	32.46	108.01	0.56	cell membrane	9	238.25026
*EcSUT2*	ELECO.r07.5AG0399930.1	5A:57109420..57116653 reverse	497	53.22	8.94	37.32	108.17	0.508	cell membrane	12	258.85469
*EcSUT3*	ELECO.r07.2AG0123760.1	2A:36651761..36655268 forward	506	53.13	8.32	39.91	98.3	0.609	cell membrane	10	222.26235
*EcSUT4*	ELECO.r07.2AG0151300.1	2A:60955893..60959784 forward	602	64.09	5.91	36.87	94.17	0.29	cell membrane	11	245.9
*EcSUT5*	ELECO.r07.2AG0135040.1	2A:49640439..49643116 reverse	529	55.83	8.29	28.18	102.74	0.516	cell membrane	12	256.70999

*NHXs* with their accession IDs (from Phytozome database), Chromosomal location and strand, size, Iso-electric point (pI), molecular weight (MW) (kDa), instabilty index, aliphatic Index, GRAVY, sub-cellular localization and transmembrane helixes (TMH).

**Table 2:** Phosphorylation sites/kinases identified in the EcSUTs.

**Protein name**	**Phosphorylation sites**			**PKA**	**cdc2**	**p38MAPK**	**cdk5**	**DNAPK**	**PKG**	**PKC**	**EGFR**	**INSR**	**CKII**	**CKI**	**GSK3**	**ATM**	**PKB**	**unsp**
	**S**	**Th**	**Ty**															
EcSUT1	18	7	2	4	8	1	0	0	0	9	0	0	2	2	0	0	0	9
EcSUT2	20	14	3	10	7	3	3	1	2	10	1	2	3	1	1	1	1	12
EcSUT3	24	10	1	3	10	1	0	0	2	17	0	0	4	3	0	0	0	12
EcSUT4	37	13	5	8	10	1	1	0	0	15	1	3	5	3	0	0	0	31
EcSUT5	26	11	3	6	8	3	2	1	1	9	0	1	2	3	3	1	0	18

PKC; protein kinase C, CK2; Casein kinase ^b^, RSK; Ribosomal S6 kinase, PKA; Protein kinase A, UNSP; Un secified phosphorylation, EGFR; Epidermal growth factor receptor, INSR; Insulin receptor precursor, PKG; Protein kinase G, CK1; Casein kinase ^a^, DNAPK; DNA dependent protein kinase, CDC2; Cell Division cycle protein ^b^, P38MAPK; P38 Mitogen activated protein kinase, CDK5; Cyclin dependant kinase ^e^, GSK3; Glycogen synthase kinase ^c^, ATM; Ataxia telangiectasia mutated, S; Serine, Th; Threonine, Ty; Tyrosine.

### 3.2 Comparative analysis of *EcSUT* genes: phylogeny, gene structure, and conserved motifs

**Fig. 1:**
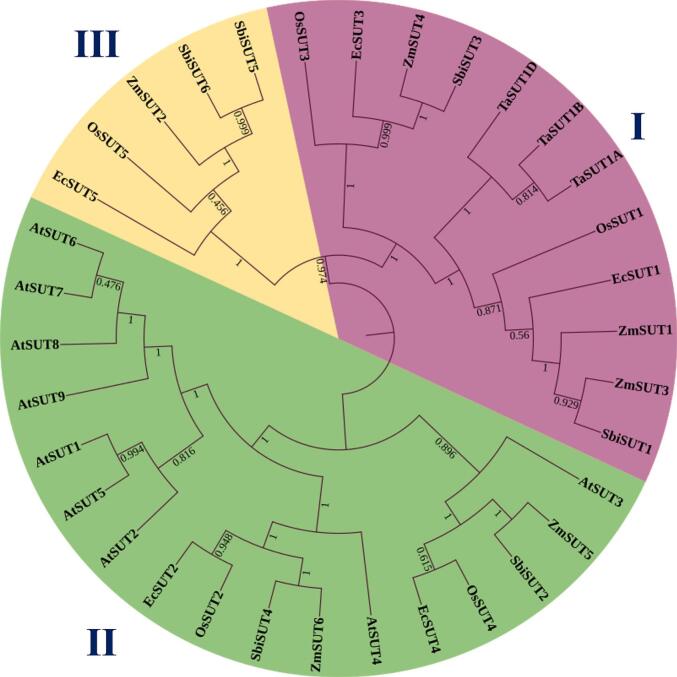
**Phylogenetic analysis of sucrose transporter (SUT) proteins from *E. coracana* and related species.** The maximum likelihood phylogenetic tree was constructed using full-length SUT protein sequences from *E. coracana* (*EcSUTs*), *Oryza sativa* (*OsSUTs*), *Zea mays* (*ZmSUTs*), *Arabidopsis thaliana* (*AtSUTs*), *Sorghum bicolor* (*SbSUTs*), and *Triticum aestivum* (*TaSUTs*). The tree clusters the SUT proteins into three major groups (I, II, and III), each highlighted in different colors.

**Fig. 2:**
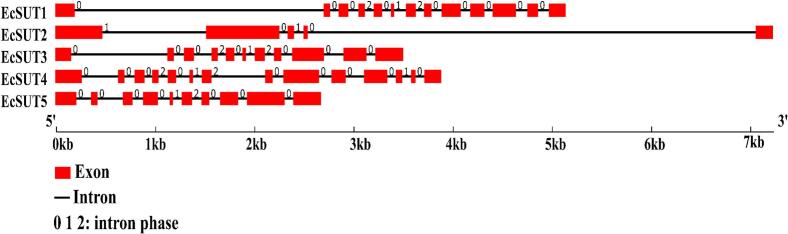
**Exon–intron organization of *EcSUT* genes in *E. coracana*.** Gene structures were visualized to show exon (red boxes), intron (black lines), and untranslated regions (UTRs, not shown). The length of each gene is scaled according to the base-pair scale (kb). Numbers above introns indicate intron phase (0, 1, or 2).

**Fig. 3:**
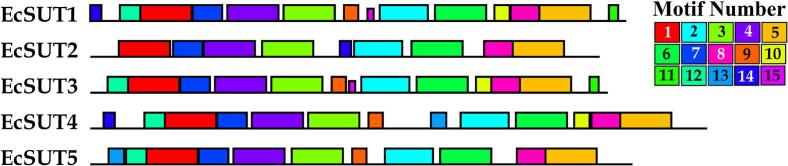
**Conserved motif distribution among EcSUT proteins.** Fifteen distinct conserved motifs (color-coded and numbered) were identified in EcSUT proteins using MEME analysis. While several motifs are shared across all members, the arrangement and presence/absence of specific motifs vary, indicating functional specialization.

To investigate the evolutionary relationships of sucrose transporter proteins in finger millet, a phylogenetic tree was constructed using full-length amino acid sequences of SUT family members from Arabidopsis, rice, sorghum, maize, and wheat. The resulting circular tree revealed that finger millet SUT proteins were distributed across all three clades, indicating functional diversification. EcSUT1 and EcSUT3 clusters within the Type I group alongside OsSUT1, ZmSUT1, SbiSUT1, and wheat orthologs (TaSUT1A, TaSUT1B). EcSUT2 and EcSUT4 belong to the Type II group with AtSUT2, AtSUT4, OsSUT2, and other orthologs from sorghum and maize (Fig. 1). Notably, EcSUT5 is part of the Type III group with OsSUT5, SbSUT5, and ZmSUT6. The analysis of gene structure showed a variety in the number and arrangement of exons and introns. *EcSUT1* has the most exons and the longest stretch of DNA, while *EcSUT3* is more compact with fewer introns (Fig. 2). The phases of the introns differ among the genes, indicating complex splicing patterns. In addition, analysis of conserved motifs identified 15 unique motifs, with key motifs present in all five proteins. This indicates that they have important functional components. However, there was noticeable variation in the arrangement of motifs. *EcSUT2* had a slightly different motif profile, suggesting possible differences in its functional roles or regulatory interactions (Fig. 3 and Table 3).

**Table 3:** Multilevel consensus sequences of motifs in *EcSUTs.*

**MOTIF #**	**SEQUENCE**	**E-VALUE**	**WIDTH**
**1**	CGIVMSHYTWHNVTESSRITTKHAFATLSFIAETFJFLYVGMDALDIEKW	6.80E-121	50
**2**	FSEDLFFIYLLPPIIFNAGFQVKKKQFFRNFMTITLFGAVG	1.60E-86	41
**3**	TDSVCTLQVLNQDETPLLYSLVFGEGVVNDATSVVLFNAIZ	8.10E-85	41
**4**	ISFKQQVIIWWAGLMRGAVSIALAYNKFTRSGHTEVPGNAIMITSTITVV	3.70E-88	50
**5**	YVIKKLYFGRHSTDREVALMMLMAYLSYM	2.30E-56	29
**6**	NLFVALLCACIVLGHLLEENRWMNESITALJIGLGTG	1.90E-55	37
**7**	RSVHYYWRKFDDSFMRPVFGGRGFV	5.50E-45	25
**8**	SPGKSIAJSSIILGLVLVGRAAFVFPLSFLSNLSKKGPLEK	1.10E-23	41
**9**	ISLGAYGLFSKLBIGTLELGDYLAIGAIF	1.90E-16	29
**10**	LFSTMVFGMLTKPLISLLIPP	3.60E-13	21
**11**	AELLDLSGILTVFF	2.50E-10	14
**12**	GNFLYLFLTSTFLGV	1.60E-05	15
**13**	PFVPGSPTERSVP	2.00E-03	13
**14**	ILLVSKGKNSH	6.10E + 03	11
**15**	RPDSLRMLLTRPT	2.00E + 05	13

### 3.3 Tertiary structure prediction and structural homology assessment

**Fig. 4:**
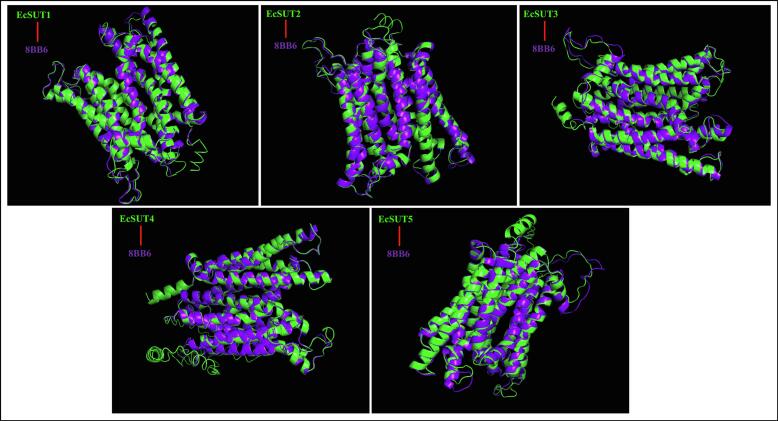
**Structural alignment of EcSUT proteins with the template 8BB6.** Comparative modeling of EcSUT1–EcSUT5 was conducted using the 8BB6 crystal structure as a reference. Predicted tertiary structures (green) were aligned with the template (purple) to assess topological conservation and transmembrane domain organization.

The three-dimensional structures of five *E. coracana* sucrose transporter proteins (EcSUT1–EcSUT5) were predicted using I-TASSER, which integrates LOMETS threading and iterative fragment assembly to generate reliable structural models. Predicted models were assessed using C-score, TM-score, and RMSD measuring the model’s accuracy to the known structure. The C-scores showed moderate to low confidence with a range between −0.12 to −2.48, where EcSUT5 had the highest model reliability (Table 4). Protein folding TM-scores ranging from 0.43 to 0.70 also had EcSUT5 scoring the highest. All five EcSUT proteins had significant structural alignment with known sucrose transporter crystal structure 8BB6A, which is demonstrated by the TM-scores of 0.783 to 0.940 and high alignment coverage of over (Cov > 0.78) (Table 4). Structure alignment performed in PyMOL showed all EcSUTs have a conserved arrangement of transmembrane helices. Notably, EcSUT2 and EcSUT5 exhibited the closest resemblance to the template structure with low RMSD values (0.76Å and 0.99 Å, respectively), suggesting identical tertiary structures essential for transporter function (Fig. 4).

**Table 4:** Structural dependent modeling parameters for the EcSUT proteins.

**Protein**	**C-Score**	**TM-Score**	**RMSD (Å)**	**Best Identified Structural Analogs in PDB**				
				**PDB Hit**	**TM-score**	**RMSD^a^**	**IDEN^a^**	**Cov**
EcSUT1	−0.87	0.60 ± 0.14	9.4 ± 4.6	8bb6A	0.888	1.20	0.437	0.906
EcSUT2	−0.37	0.67 ± 0.13	8.1 ± 4.4	8bb6A	0.940	0.76	0.497	0.948
EcSUT3	−1.10	0.58 ± 0.14	10.0 ± 4.6	8bb6A	0.874	1.43	0.411	0.896
EcSUT4	−2.48	0.43 ± 0.14	13.9 ± 3.9	8bb6A	0.783	0.53	0.457	0.786
EcSUT5	−0.12	0.70 ± 0.12	7.6 ± 4.3	8bb6A	0.916	0.99	0.422	0.929

### 3.4 Synteny analysis of SUT genes in *E. coracana* and related grasses

**Fig. 5:**
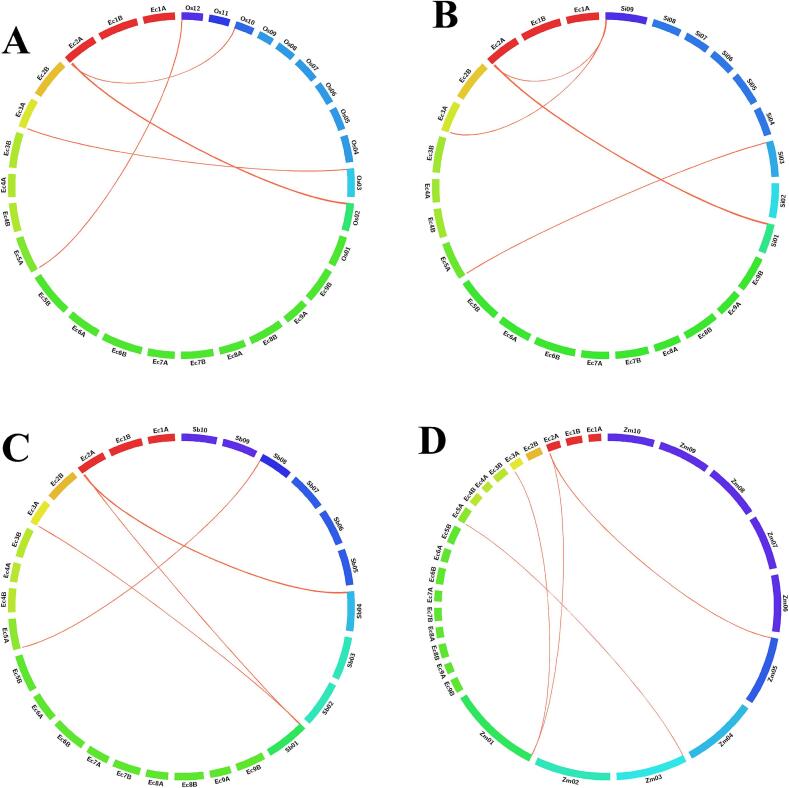
**Syntenic relationships between *EcSUT* genes in *E. coracana* and their orthologs in four grass species**. Circos plots illustrate conserved chromosomal regions linking *E. coracana* SUT genes with orthologous loci in *O. sativa*, *S. bicolor*, *S. italica*, and *Z. mays*. Colored ribbons represent collinear gene pairs, highlighting conserved synteny and potential ancestral genomic blocks across the Poaceae family.

To examine the evolutionary conservation of sucrose transporter (*SUT*) genes, a synteny analysis was carried out between *E. coracana* and four grass species: *O. sativa*, *S. bicolor*, *S. italica*, and *Z. mays*. All five *EcSUT* genes (*EcSUT1*–*EcSUT*5) displayed clear orthologous relationships with *SUT* genes in the compared genomes (Fig. 5A-D). In *O. sativa*, *EcSUT2* showed the highest sequence identity (94 %) with *OsSUT2*, followed by *EcSUT1* (87 %) and *EcSUT4* (86 %). Both *EcSUT4* and *EcSUT5* were syntenic with genes located on rice chromosome 2, indicating conserved genomic regions (Fig. 5A). In *S. italica*, each *EcSUT* gene had a corresponding ortholog, with identity values between 83 % and 93 %. The *EcSUT2*-*SiSUT2* pair exhibited the highest similarity (93 %), and *EcSUT1* and *EcSUT3* corresponded to genes on chromosome 9, suggesting potential clustering in this region (Fig. 5B). In *S. bicolor*, all *EcSUT* genes had orthologs with identity levels ranging from 79 % (*EcSUT5*) to 91 % (*EcSUT2*). *EcSUT1* and *EcSUT3* were both aligned to chromosome 1, suggesting conservation of gene order (Fig. 5C). In *Z. mays*, synteny was observed for all five *EcSUTs*, with identity values ranging from 75 % (*EcSUT5*) to 91 % (*EcSUT2*). The maize orthologs were located on chromosomes 1, 3, and 5, reflecting the species complex and rearranged genome structure (Fig. 5D).

### 3.5 Protein-protein interaction (PPI) network analysis

**Fig. 6:**
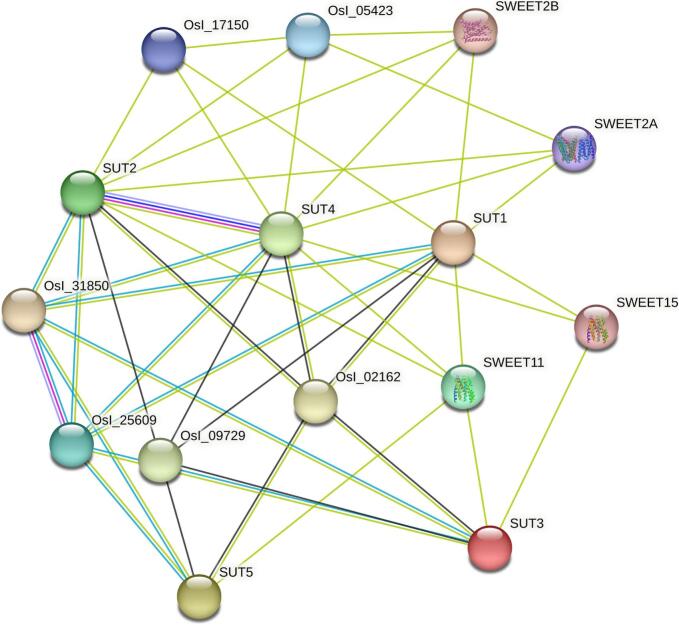
**Protein–protein interaction (PPI) network of EcSUT orthologs predicted by STRING.** The interaction map illustrates functional associations among sucrose transporter homologs and sugar transport-related proteins.

Protein-protein interaction (PPI) network was constructed with the STRING database to analyze the functional relationships between transporters and associated proteins. As shown in the resulting network, several key interactions among sucrose transporters (SUTs) and sugar efflux transporters (SWEETs) are complex regulatory framework of sugar transport and allocation (Fig. 6). Notably, EcSUT2 emerged as a central hub with strong predicted interactions with SWEET2A, OsI_05423, OsI_17150, and EcSUT4, suggesting that EcSUT2 performs an integrative role in coordinating sucrose uptake and distribution. Furthermore, SUT4 showed extensive interactions with SWEET2A, SWEET15, and other transporters, which indicates the functional versatility of EcSUT4 (Fig. 6). The presence of multiple SWEET family proteins (e.g., SWEET2A, SWEET11, and SWEET15) within the network illustrates the potential collaboration between SUT and SWEET transporters in maintaining sugar homeostasis. These interactions may underpin coordinated sugar efflux and retrieval for phloem loading, unloading, and intercellular transport, driven by diverse physiological conditions (Fig. 6).

### 3.6 *Cis*-regulatory element analysis of *EcSUT* promoters

**Fig. 7:**
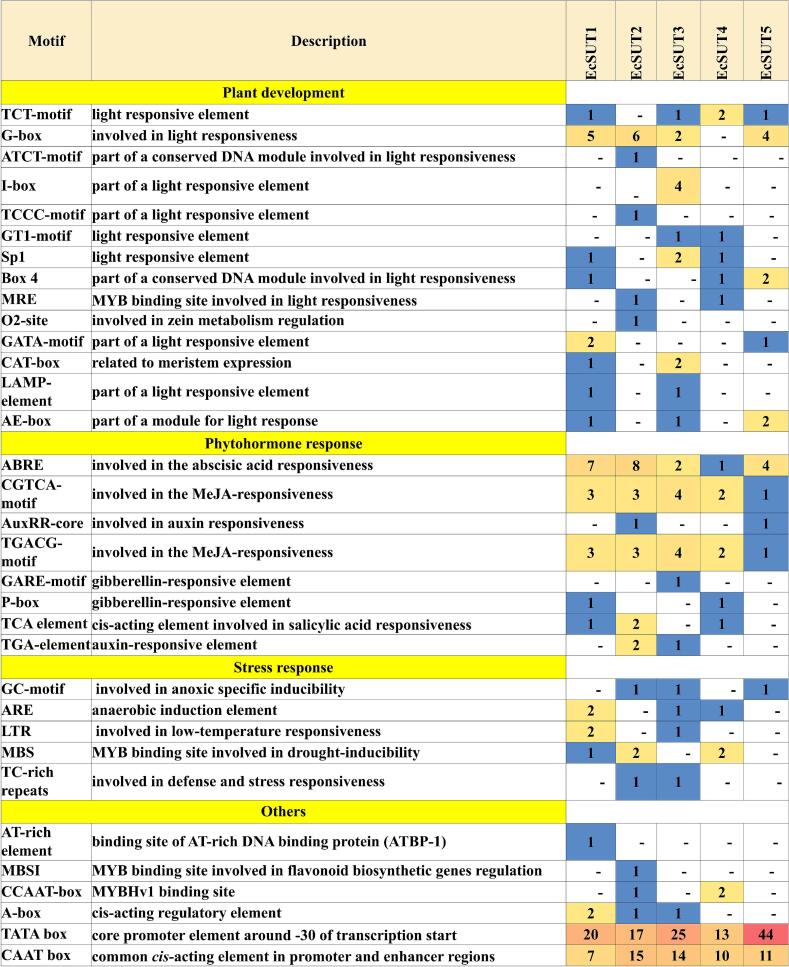
**Predicted *cis*-regulatory elements in the promoter regions of *EcSUT* genes.** The heatmap displays the distribution of *cis*-elements associated with plant development, phytohormone signaling, stress response, and other regulatory processes within 1.5 kb upstream regions of *EcSUT1*–*EcSUT5*. Color gradients represent the relative abundance of each element, highlighting differences in potential regulatory mechanisms among the gene family members.

In finger millet, *cis*-regulatory element analysis of five *EcSUT* genes showed a unique set of motifs linked to plant development, hormonal signaling, and stress response pathways. Elements involved in light responsiveness such as the G-box, TCT-motif, and I-box were particularly abundant in *EcSUT1*, *EcSUT3*, and *EcSUT5*, suggesting that these genes may be responsive to light signals and play roles in processes linked to photosynthesis and growth. Several hormone-responsive motifs were also identified, including ABRE (abscisic acid), CGTCA- and TGACG-motifs (methyl jasmonate), and the TCA-element (salicylic acid) (Fig. 7). These elements were most frequently found in *EcSUT1*, *EcSUT2*, and *EcSUT4*, pointing to possible regulation of these genes by hormonal signaling during stress adaptation. Notably, *EcSUT2* had the highest number of hormone- and stress-associated elements including LTR (low temperature), ARE (anaerobic conditions), and MBS (drought response) indicating its potential involvement in multiple abiotic stress pathways (Fig. 7). All the promoters contained the fundamental core elements TATA- and CAAT-boxes, with *EcSUT5* showing the highest number of TATA-boxes (44), which may be linked to higher transcriptional potential (Fig. 7). These findings highlight the varied regulatory potential of *EcSUT* promoters and their possible roles in developmental processes, hormonal cross-talks, and environmental stress adaptation.

### 3.7 Expression profiling of *EcSUT g*enes under drought stress

**Fig. 8:**
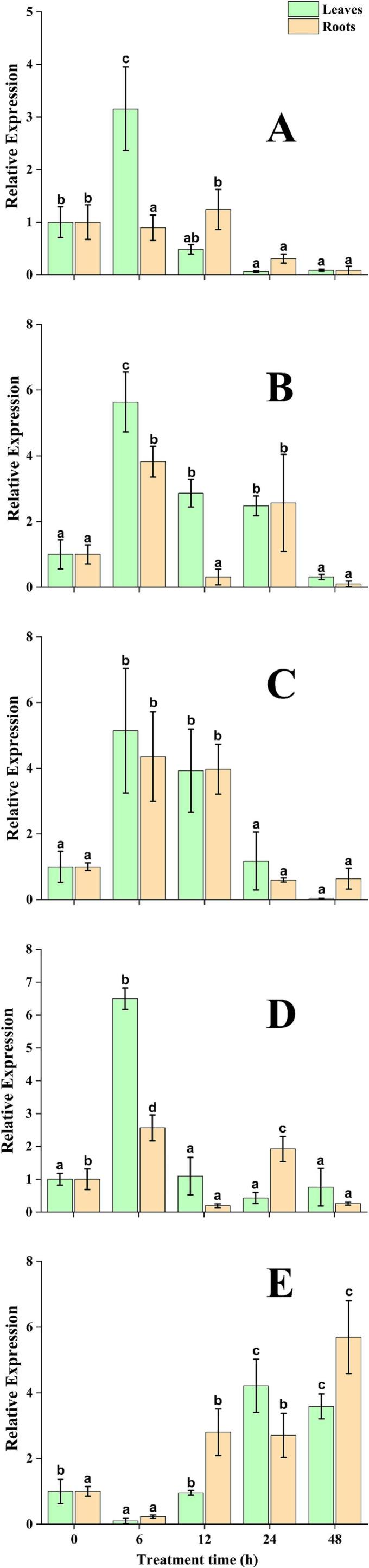
The expression patterns of *EcSUT1* to *EcSUT5* were examined in the leaves and roots of finger millet subjected to drought stress (20 % PEG) at five time points: 0, 6, 12, 24, and 48 h. Quantitative RT-PCR was used to measure relative gene expression, with normalization against a reference gene and calculations performed using the 2^−ΔΔ^Ct method. Expression data are shown as bar plots, displaying the mean ± standard error from three independent biological replicates. A) *EcSUT1*, B) *EcSUT2*, C) *EcSUT3*, D) *EcSUT4* and E) *EcSUT5*.

The expression dynamics of five *EcSUT* genes were evaluated in the leaves and roots of finger millet in response to drought stress over five-time intervals (0, 6, 12, 24, and 48 h). Chord diagrams were used to visualize gene expression dynamics separately for each tissue type (Fig. 8A and B). In leaf tissue, *EcSUT2*, *EcSUT3*, and *EcSUT4* showed marked upregulation at 6 h, indicating an early response to drought stress. In contrast, *EcSUT5* expression increased at 24 and 48 h, reflecting a delayed activation during prolonged stress conditions (Fig. 8A). In root tissue, elevated expression of *EcSUT3* and *EcSUT5* was observed between 12 and 48 h, suggesting their involvement in long-term stress response mechanisms. EcSUT1 showed moderate induction during the early phase (6–12 h) (Fig. 8B). Among the five genes, *EcSUT2* displayed variable expression across both tissues and time points, suggesting it may play a role in both early and late stages of the stress response. Overall, the data highlight tissue-specific and time-dependent regulation of *EcSUT* genes under drought stress conditions in finger millet.

## 4 Discussion

The identification of five sucrose transporter (*EcSUT)* genes in finger millet highlights the molecular diversity and complexity of sucrose transport mechanisms in this underutilized cereal. The predicted EcSUT proteins contain 9–12 transmembrane helices, consistent with the Arabidopsis,^11^ rice,^4^ sorghum ^32^, and wheat ^33^. The presence of multiple transmembrane domains ensures their functionality in facilitating sucrose movement across lipid bilayers, a fundamental process in source-to-sink transport during plant growth and stress adaptation. Chromosomal localization analysis revealed that the *EcSUT* genes are distributed on chromosomes 2A, 3A, and 5A, indicative of potential segmental duplications or evolutionary gene rearrangements that may have contributed to the expansion of the SUT family in *E. coracana*. Phosphorylation site analysis revealed EcSUT4 had the highest number of predicted regulatory sites, especially for PKC. At the same time, cdc2 and CKII motifs were conserved across all proteins, indicating potential involvement in stress signaling and metabolic regulation. The abundance of unspecified kinase sites in EcSUT4 and EcSUT5 points to their complex regulation, possibly linked to stress adaptation. These findings suggest functional diversification among EcSUTs, supporting *E. coracana's* ability to adapt to challenging environmental conditions.

Phylogenetic analysis revealed that finger millet SUTs are distributed across all three major clades (Groups I–III), reflecting functional diversification consistent with other monocots (*O. sativa*), maize (*Z. mays*), and sorghum (*S. bicolor*), as well as the dicot model *A. thaliana*. *EcSUT1* and *EcSUT3*, are closely related to sucrose transporters such as *OsSUT1*, *ZmSUT1*, and *SbSUT1*. These plasma membrane-localized transporters are known for their high-affinity sucrose transport and play critical roles in phloem loading and long-distance translocation of photo-assimilates (Scofield et al., 2007).^4^ In rice and wheat, SUT1-type transporters are upregulated during grain development, indicating a major role in sucrose delivery to sink tissues.^4^, ^5^ EcSUT2 and EcSUT4 clustering with OsSUT2, AtSUT2, AtSUT4, which are tonoplast-localized transporters involved in vacuolar loading or retrieval. For instance, OsSUT2 and AtSUT4 are tonoplast-localized and function in sucrose efflux from vacuoles, contributing to intracellular sugar homeostasis.^34^ EcSUT5, includes orthologs such as OsSUT5 and ZmSUT6, which are less studied but are believed to have unique physiological roles.

Synteny analysis confirmed that the SUT gene family in *E. coracana* is conserved across major grass species, with each *EcSUT* gene having a corresponding ortholog in rice, sorghum, foxtail millet, and maize. Among these, *EcSUT2* showed the highest sequence similarity across species, indicating it likely plays a fundamental and conserved role in sucrose transport in grasses.^4^, ^35^ Its strong conservation suggests it is under purifying selection, possibly due to its involvement in critical functions such as phloem loading or carbon partitioning^32^. In contrast, *EcSUT5*, although less conserved (75–83 % identity), stands out due to its complex promoter structure and marked upregulation under drought conditions. This divergence in sequence and regulatory regions may reflect adaptive changes, allowing it to respond more effectively to environmental stress. The presence of *EcSUT1* and *EcSUT3* orthologs on the identical chromosomes in *S. italica* and *S. bicolor* further points to possible segmental duplication events or conserved chromosomal arrangements inherited from a common ancestor.

Tertiary structure modeling and comparative analysis using the Arabidopsis AtSUT1 crystal structure (PDB 8BB6A) as a template, homology models confirmed that all EcSUT proteins share the characteristic sucrose‑transporter fold. The close resemblance of EcSUT2 and EcSUT5 to the template, indicated by favorable TM-scores and low RMSD values, highlights their conformational stability and possible evolutionary conservation of transport function. Protein-protein interaction analysis revealed SUT2 and SUT4 as major players in finger millet’s sugar transport system. The interaction between EcSUT2 and both SWEET2A and SWEET11 are worth noting. These SWEET proteins have been previously linked to key processes like phloem loading mechanism, plants use to distribute sugars from source to sink and also play roles during stress responses.^36^, ^37^

Promoter analysis also provides evidence for the complex regulation of EcSUTs. The presence of hormone-responsive elements such as ABRE (linked to abscisic acid), MeJA-responsive motifs, and TCA elements (associated with salicylic acid) indicates potential involvement in hormonal cross-talk under stress. Interestingly, EcSUT3 and EcSUT5 showed more stress-related elements such as ARE (linked to oxidative stress) and MBS (involved in drought response), pointing to their possible roles in helping the plant cope with environmental challenges. This *cis*-regulatory variation provides a transcriptional basis for the differential expression patterns that develop under drought stress.

The expression of *EcSUT* genes in finger millet varies across tissues and time in response to drought stress caused by 20 % PEG treatment. In the leaf tissue, *EcSUT2*, *EcSUT3*, and *EcSUT4* responded rapidly, with increased expression observed as early as 6 h after treatment. This suggests that these genes may play a role in the initial stress response, possibly by facilitating rapid sucrose redistribution. On the other hand, *EcSUT5* showed a delayed response, with higher expression at 24 and 48 h, pointing to a function during prolonged stress exposure. This pattern indicates that *EcSUT5* may be critical for long-term drought adaptation by remobilizing stored sucrose to maintain root metabolism, osmotic balance, and turgor, thereby supporting continued water and nutrient uptake. *EcSUT3* and *EcSUT5* showed elevated expression in the roots during the later stages (12–48 h), which may support the root systems metabolic activity or stress recovery processes. *EcSUT1* was moderately expressed during the early time points (6 and 12 h), hinting at a possible role in early root response. Among all the genes studied, *EcSUT2* stood out due to its changing expression across both tissues and over time, suggesting that it may serve multiple roles depending on the stage of stress. This sustained response hints at a possible role in long-term stress adaptation, perhaps by ensuring a steady supply of sucrose to maintain root metabolism and growth under prolonged drought.

Interestingly, similar patterns of SUT gene expression in response to abiotic stress have been reported in different crop species. In tomato, the upregulation of *SlSUT1* during drought and salinity postpones leaf senescence and enhances stress tolerance,^38^ but in tobacco, the expression of *NtSUT1* increases under water deficit and salt to facilitate sucrose transfer to the roots.^39^ . In wheat, *TaSUT1* and *TaSUT2* are synthesised during grain filling under situations of water scarcity.^5^ These resemblances suggest that finger millet may share conserved strategies with other plants, using a coordinated network of sucrose transporters to manage immediate and long-term responses to drought.

The co-ordinated expression of *EcSUTs* under PEG-induced drought stress suggests that these proteins help with stress adaptation by maintaining energy supply and osmotic balance in roots. *EcSUTs* play a crucial role in redistributing sugars produced during photosynthesis from the shoots to the roots under drought stress. This process supports roots maintain metabolism, turgor and water absorption even when shoot growth slows. This mechanism may be attributed to the drought resistance of finger millet plants. Similar stress-induced upregulation of SUT genes has been reported in rice, tomato and tobacco, where improved sucrose export is linked with delayed leaf ageing and better stress resistance.^38^ Therefore, the drought-induced expression of *EcSUTs* represents a conserved adaptive strategy in which sucrose transport directly supports drought tolerance. Expanding the SUT gene family enhances finger millet's resilience to drought by generating paralogs with distinct regulatory mechanisms and functions. This diversity enables plants to optimise sucrose transport across tissues during stress, maintaining osmotic equilibrium and a consistent energy source. This adaptability in carbon distribution offers a distinct evolutionary benefit, facilitating growth and survival during drought conditions.

## 5 Conclusion

The five *EcSUT* genes of finger millet show evolutionary conservation of all major phylogenetic classes with distinct structural and regulatory features. Synteny analysis revealed a high sequence conservation, especially for *EcSUT2* (91–94 % identity) for the grass species. The protein–protein interaction network positions EcSUT2 as a central hub for coordination with SWEET transporters, and promoter analysis reveals various regulatory mechanisms, with EcSUT2 having the most stress-responsive elements. In drought conditions, *EcSUT1*-*EcSUT4* showed rapid and temporary responses that peaked in 6–12 h, while EcSUT5 showed constant upregulation that peaked in 48 h, especially in the roots. This temporal expression pattern indicates that *EcSUT5* plays a crucial role in long-term drought adaptation, and it is considered a candidate for improving drought resistance to cereal crops through breeding programmes.

## CRediT authorship contribution statement

**Kasinathan Rakkammal:** Writing – original draft, Visualization, Validation, Software, Methodology, Investigation, Conceptualization. **Pandiyan Muthuramalingam:** Writing – review & editing, Visualization, Validation, Software, Resources, Methodology, Investigation, Data curation. **Hyunsuk Shin:** Writing – review & editing, Validation, Software. **Manikandan Ramesh:** Writing – review & editing, Validation, Supervision, Software, Resources, Methodology, Investigation, Funding acquisition, Conceptualization.

## Declaration of competing interest

The authors declare that they have no known competing financial interests or personal relationships that could have appeared to influence the work reported in this paper.
