# Supplementary material for: Genome-wide identification and evolutionary analysis of SUT genes reveals key regulators of drought stress response in finger millet (Eleusine coracana)
Source: J Genet Eng Biotechnol. 2025 Oct 14;23(4):100592. doi: 10.1016/j.jgeb.2025.100592 (PMC12550293; doi:10.1016/j.jgeb.2025.100592)
Supplement: Supplementary Data 1 [file mmc1.docx]

**Table S1.** Details of *EcSUT* Primers for qRT-PCR analysis

| **S. No** | **Gene Name** | **F Primer** | **R primer** | **Product size** | **Tm**  **(ºC)** |
| --- | --- | --- | --- | --- | --- |
| 1. | *EcEF1α* | CCTGGTGATAATGTGGGATTC | GGACAGATCGGCAATGGCTA | 161 | 59.4 |
| 2. | *EcSUT1* | TGCCCACCTCAACACTTCA | AGGAATGTGCAGGGAGGTC | 105 | 59.1 |
| 3. | *EcSUT2* | GCTCAGATTGCGGCTTTCA | GAACAGAGTCGGGACAGCT | 102 | 59.0 |
| 4. | *EcSUT3* | AGTGCCATACAACTGCCAAA | CGGTTCTGGAAATCTACCCG | 130 | 58.0 |
| 5. | *EcSUT4* | TGTGGCTGTGTGGACCTATT | GTCACTGTAAAGACCGACGC | 164 | 58.9 |
| 6. | *EcSUT5* | ATCAGCATCGTCCGTCTCTT | ACAAACGACATGAACACGCA | 188 | 58.8 |
